# Mitochondrial replacement approaches: challenges for clinical implementation

**DOI:** 10.1186/s13073-016-0380-2

**Published:** 2016-11-25

**Authors:** Thomas Klopstock, Barbara Klopstock, Holger Prokisch

**Affiliations:** 1 Department of Neurology, Friedrich-Baur-Institute, Ludwig-Maximilians-University, Munich, 80336 Germany; 2 Munich Cluster for Systems Neurology (SyNergy), Munich, 80336 Germany; 3 German Center for Neurodegenerative Diseases (DZNE), Munich, 80336 Germany; 4 Fachhochschule für Öffentliche Verwaltung und Rechtspflege in Bayern (FHVR), Wasserburg, 83512 Germany; 5 Institute of Human Genetics, Technical University Munich, Munich, 81675 Germany; 6 Institute of Human Genetics, Helmholtz Zentrum Munich, Neuherberg, 85764 Germany

## Abstract

The advent of mitochondrial replacement techniques poses many scientific, regulatory, and ethical questions. Previous studies suggest good safety and efficacy profiles of these techniques, but challenges remain for clinical implementation and international consensus is needed on the regulation of these approaches.

## Mitochondrial disorders and their genetic background

Mitochondrial disorders are a heterogeneous group of rare inherited diseases of energy metabolism that range in severity from fatal childhood-onset to mild late-onset syndromes. There are more than 1500 mitochondrial proteins, most of which are nuclear-encoded, with only 13 encoded by mitochondrial DNA (mtDNA). Genetic counseling and prenatal or preimplantation diagnostics are available options for nuclear gene defects as the mutations segregate in a Mendelian way (mostly autosomal recessive). However, the situation is much more complicated for mtDNA mutations, which are exclusively transmitted through the maternal line. As the mutation load might differ from cell to cell and from tissue to tissue, recurrence risks are difficult to estimate and prenatal and preimplantation genetic diagnoses are unreliable. Mitochondrial replacement techniques (MRTs) could be used to enable women who carry mtDNA mutations to have a genetically related child with a greatly reduced risk of mtDNA disease.

## Mitochondrial replacement

Two mitochondrial replacement techniques have been developed, maternal spindle transfer and pronuclear transfer. In maternal spindle transfer, the chromosome spindle apparatus of the carrier mother is removed from her unfertilized oocyte and inserted into the donor mother’s unfertilized and enucleated oocyte that contains normal mtDNA. The hybrid oocyte is then fertilized in vitro by the father’s sperm and implanted into the carrier mother by standard in vitro fertilization (IVF) procedures. In pronuclear transfer, both carrier mother’s and donor mother’s oocytes are first fertilized in vitro. The carrier mother’s pronucleus is inserted into the donor mother’s enucleated oocyte containing normal mtDNA, and the embryo is implanted into the carrier mother by IVF. The mitochondrial replacement approach is generic; instead of targeting a specific mutation, MRTs replace nearly all mitochondria and their resident mtDNA and so could be applied to any inherited mtDNA disease.

## Safety and efficacy

The technical feasibility of MRTs has been demonstrated in animal models, including primates, and apparently healthy offspring have been born. One of the main concerns is that some carry-over of the maternal mtDNA is unavoidable, which means that a few thousand mtDNA copies of the carrier mother (a percentage of them mutant) are introduced into the donor mother’s egg (which contains several hundred thousand copies of a different but normal mtDNA). Although the resulting proportion of mutant mtDNA might be fairly low initially, this could change rapidly within a single generation. Therefore, leakage of mutant mtDNA could still lead to disease in the resulting offspring and in future generations. A model of mtDNA inheritance showed that the chance of disease recurrence in subsequent generations could be reduced dramatically by lowering the proportion of mutant mtDNA to less than 5% [1]. Indeed, optimization of the pronuclear transfer protocol in early preclinical studies reduced mtDNA carryover to less than 2% heteroplasmy in the majority (79%) of blastocysts and none had more than 5% heteroplasmy [2]. In conclusion, MRT can markedly reduce the risk of mtDNA disease but cannot guarantee prevention. The same study [2] showed that refinements in the protocol, which included pronuclear transfer shortly after completion of meiosis, promoted efficient development to good quality blastocysts with improved survival. Importantly, gene expression patterns and incidence of aneuploidy were the same for these blastocysts and controls.

Another safety issue that is currently debated is the possible incompatibility between mtDNA and nuclear DNA if originating from different mothers. Reciprocal mitochondrial replacement in zygotes between widely divergent mouse lines revealed an interspecies reproductive barrier as a result of functional incompatibility between mitochondrial and nuclear genomes [3]. However, intraspecies mitochondrial replacement in mice and non-human primates resulted in viable fertile offspring that were indistinguishable from controls, with normal growth and development to adults [4]. Likewise, mitochondrial replacement resulted in no apparent abnormalities in human embryos and embryonic stem cells with unmatched donor mtDNA [5]. In light of the comparably low sequence difference even between human mtDNA haplotypes of distantly related populations, incompatibilities between mtDNA and the nuclear genome are unlikely among human populations. Nevertheless, matching donor and recipient mtDNA haplotypes in clinical mitochondrial replacement might still be judicious, but might be limited by donor egg availability.

## The current status of clinical implementation and regulation

Legislation in most countries prohibits germline modification procedures owing to safety concerns and ethical and societal issues (Box 1). However, should mitochondrial replacement be considered a form of human germline modification? Some say it is because the procedures alter the genetic content of human oocytes or embryos which is transmitted to future generations in the case of female offspring. Others say mitochondrial replacement involves only an exchange of mtDNA without modification, thus being a kind of organelle transplantation, analogous to organ transplantations later in life. Undoubtedly, a restriction of mitochondrial replacement to male offspring would circumvent the germline modification issue, as this would preclude transmission of the donor mtDNA to future generations.

Accordingly, mitochondrial replacement has led to considerable divergence in global policy [6]. To date, the UK and USA are the only countries moving forward with this technology. In the UK, after years of constructive discussions and consultations guided by the Human Fertilization and Embryology Authority (HFEA), Parliament approved the licensed clinical use of both spindle and pronuclear transfer in October 2015. Clinical application is imminent, subject firstly to HFEA’s implementation of a licensing framework for mitochondrial replacement clinics, and secondly to three outstanding pre-clinical safety and efficacy reports [6]. The first mitochondrial replacement services are expected to assess safety and efficacy in a clinical study, which will enroll ten applicants per year [7]. In the USA, a more cautionary approach has been taken: a Food and Drug Administration (FDA) expert panel concluded to further delay the approval of mitochondrial replacement until more preclinical data were available, and notably, a National Academies of Sciences expert committee recommended that mitochondrial replacement should only create male embryos to preclude the transmission of the donor mtDNA to future generations, thereby avoiding the germline modification issue [8].

However, recently a US fertility specialist circumvented US regulations and applied mitochondrial replacement in Mexico, a country without any rules in this field [9]. The procedure was conducted for a Jordanian couple who had previously lost two children to Leigh syndrome due to a mtDNA mutation and led to the birth (in April 2016) of a seemingly healthy boy. While some experts hailed the action as overdue progress in the field, there is also considerable disapproval of the circumvention of regulations by carrying out the procedure in unregulated countries [10].

## Conclusions

It seems that Pandora’s Box has been opened regarding mitochondrial replacement. Now, it is of utmost importance to avoid uncontrolled proliferation of the technique. There should be an international consensus that the first human applications are carried out in the well-regulated framework of a clinical trial in expert centers, as is the case for other first-in-man studies. Until this is successfully set up, a voluntary restriction of “solo efforts” by scientists and fertility doctors would be highly desirable. On the other hand, regulatory agencies and public debates about ethical implications and limitations need to speed up to avoid frustration of affected families and experts who are in favor of mitochondrial replacement. Recent developments have shown, however, that national legislation is not sufficient. International governance structures are needed to take decisions on the implementation of biomedical innovations. One possible forum for this could be the Biotechnology Initiative Program of the United Nations, which already has the appropriate remit to tackle complex bioethical issues [6].

## Box 1. Ethical and societal challenges

Progress in reproductive medicine poses a myriad of ethical and societal questions. Here, we give our views on two of these questions:

***Will approval of mitochondrial replacement open the door to germline manipulation?*** With technological advances in reproductive medicine, are societies prepared to allow progress, to welcome prevention and extinction of severe diseases, to concede patient autonomy on the one hand and to value, respect, and protect human life and dignity by setting limits to unrealistic expectations on the other? Previous experience indicates that the application of novel techniques is mostly driven by their feasibility, less so by the regulatory frameworks. Accordingly, we do not think that policy (approval or the lack of approval) regarding mitochondrial replacement will affect the progress of other techniques that can be used for germline manipulation, such as genome editing.

***Do children born from mitochondrial replacement have three parents, that is, two mothers?*** Mitochondrial replacement does not constitute any legal, social, or psychological component of motherhood and constitutes only a small contribution to biological motherhood. Therefore, the designation “three-parent baby”, as often used in the media, is unfortunate and should not, in our opinion, be used by medical doctors and scientists. Currently, mitochondrial replacement is mostly propagated for families with a segregation of extremely severe mtDNA-related disorders. However, if a family harboring a mtDNA mutation responsible for a relatively less severe disease such as Leber’s hereditary optic neuropathy asks for mitochondrial replacement to protect their children from an increased risk of visual deterioration, who could argue against this? These decisions cannot be taken by individual physicians but require a framework with inclusion and exclusion criteria and a multidisciplinary team including ethicists for case-by-case decisions.

## Abbreviations

FDA: Food and Drug Administration
HFEA: Human Fertilization and Embryology Authority
IVF: In vitro fertilization
mtDNA: Mitochondrial DNA
MTR: Mitochondrial replacement techniques
